# A newly developed snack effective for enhancing bone volume

**DOI:** 10.1186/1475-2891-8-30

**Published:** 2009-07-03

**Authors:** Junji Ohtani, Rene Arturo Marquez Hernandez, Hiroko Sunagawa, Tadashi Fujita, Toshitsugu Kawata, Masato Kaku, Masahide Motokawa, Natsumi Tsuka, Hiroyuki Koseki, Yayoi Matsuda, Hidetaka Hayashi, Sara Abedini, Kazuo Tanne

**Affiliations:** 1 Department of Orthodontics and Craniofacial Developmental Biology, Graduate School of Biomedical Sciences, Hiroshima University, 1-2-3, Kasumi, Minami-ku, Hiroshima-city, Hiroshima, Japan

## Abstract

**Background:**

The incidence of primary osteoporosis is higher in Japan than in USA and European countries. Recently, the importance of preventive medicine has been gradually recognized in the field of orthopaedic surgery with a concept that peak bone mass should be increased in childhood as much as possible for the prevention of osteoporosis. Under such background, we have developed a new bean snack with an aim to improve bone volume loss. In this study, we examined the effects of a newly developed snack on bone volume and density in osteoporosis model mice.

**Methods:**

Orchiectomy (ORX) and ovariectomy (OVX) were performed for C57BL/6J mice of twelve-week-old (Jackson Laboratory, Bar Harbar, ME, USA) were used in this experiment. We prepared and given three types of powder diet *e.g.*: normal calcium diet (NCD, Ca: 0.9%, Clea Japan Co., Tokyo, Japan), low calcium diet (LCD, Ca: 0.63%, Clea Japan Co.,) and special diet (SCD, Ca: 0.9%). Eighteen weeks after surgery, all the animals were sacrified and prepared for histomorphometric analysis to quantify bone density and bone mineral content.

**Results:**

As a result of histomorphometric examination, SCD was revealed to enhance bone volume irrespective of age and sex. The bone density was increased significantly in osteoporosis model mice fed the newly developmental snack as compared with the control mice. The bone mineral content was also enhanced significantly. These phenomena were revealed in both sexes.

**Conclusion:**

It is shown that the newly developed bean snack is highly effective for the improvement of bone volume loss irrespective of sex. We demonstrated that newly developmental snack supplements may be a useful preventive measure for Japanese whose bone mineral density values are less than the ideal condition.

## Background

Postmenopausal osteoporosis and hypogonadism are accompanied by serious complications such as climacteric disturbance, hyperlipidemia, coronary arterial disease, and femoral fracture, impairing the quality of life (QOL) in aged people in particular. The incidence of primary osteoporosis was 24% in Japanese females aged 50 years or older according to the diagnostic criteria established by the Japanese Society for Bone and Mineral Research[1]. This number is higher than those in USA and European countries [2,3], which has become a serious social problem.

A recent clinical report clarified that treatment aiming at the prevention of femur fracture was most effective for the maintenance and improvement of the QOL[4], attracting a marked attention toward better understanding of bone metabolism. Peak bone mass, in general, is acquired from childhood to adolescence, but 35% of cortical bone and 50% of trabecular bone are lost thereafter[5]. The importance of preventive medicine thus has been gradually recognized in the field of orthopaedic surgery with a concept that the peak bone mass is to be increased and saved during childhood as much as possible [6,7]. Such an idea has been attracting a special attention in the field of clinical medicine for the prevention of osteoporosis. The importance of normal development and growth of bone until adolescence has also been reported [8,9].

Furthermore, treatment modality for osteoporosis was examined in previous studies on sex hormones and bone metabolism using aged animal models. In these studies, estrogen deficiency was identified as a principal cause of osteoporosis [10-12]. However, there have been no established treatment methods for osteoporosis. This fact indicates that the influences of sex hormones on bone metabolism are very complicated, and the mechanism is very difficult to be understood.

A newly developed snack used in this study contains appropriate amounts of minerals (calcium and magnesium) as well as soybean isoflavone which has a sex hormone-like action. Casein phosphopeptide (CPP), recognized as a specified food for health, is also included. The objective of this study was to investigate bone metabolism in osteoporosis model mice fed the new snack and to elucidate the effects on the improvement of osteoporosis in terms of the bone volume and density.

## Materials and methods

### Experimental animals

**Figure 1:**
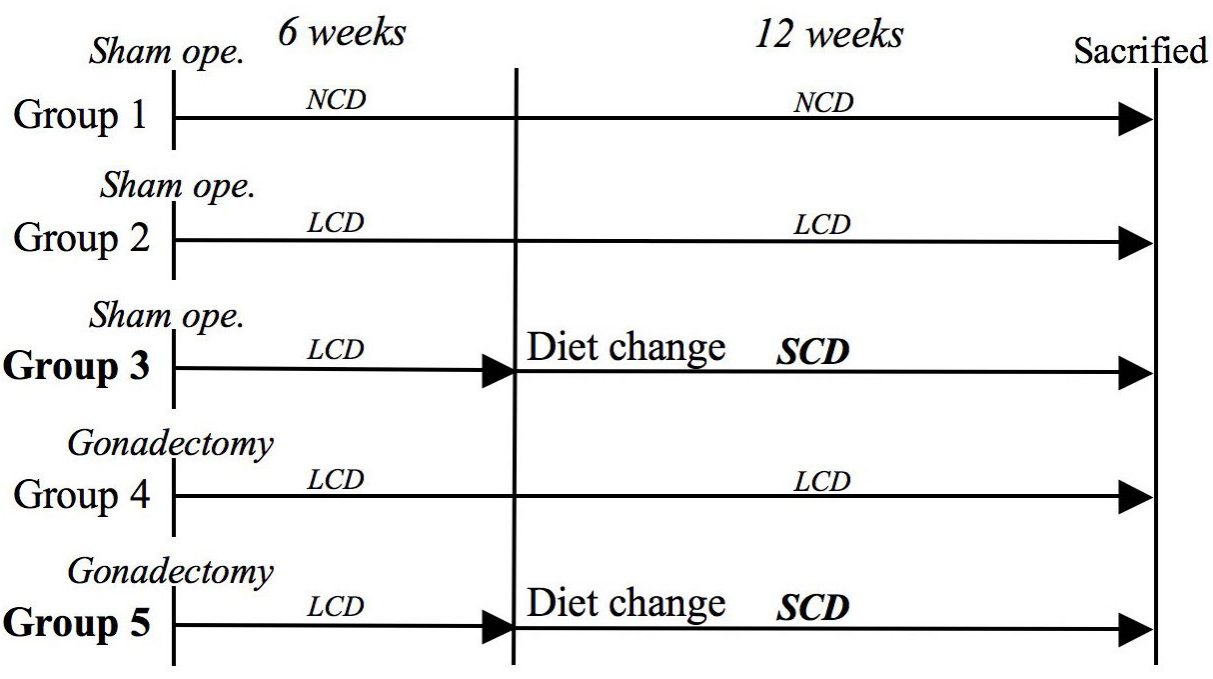
**Time schedule during experiments**.

Fifty male and fifty female C57BL/6J mice of twelve-week-old (Jackson Laboratory, Bar Harbar, ME, USA) were used in this experiment and divided respectively into five groups with ten in each sex. In the experimental groups, orchiectomy (ORX) and ovariectomy (OVX) were performed for ten mice in each gender at the beginning of experiment to create the osteoporosis model mice. ORX and OVX were performed for experimental mice by means of a stereoscopic microscope (SZX9, Olympus Optical Co., Tokyo, Japan) under general anesthesia with sodium pentobarbital, whereas the control mice underwent sham operation. The detail of time schedule during a series of experiment is shown in Figure 1. These animals were treated under the ethical regulations defined by the Ethics Committee, Hiroshima University Faculty of Dentistry.

### Supplied diet

We prepared three types of powder diet *e.g.*: normal calcium diet (NCD, Ca: 0.9%, Clea Japan Co., Tokyo, Japan), low calcium diet (LCD, Ca: 0.63%, Clea Japan Co.,) and special diet (SCD, Ca: 0.9%) developed as a new bean snack. NCD with 0.9% calcium was made on basis of objective consumption of calcium intake in humans defined by the Ministry of Health, Labour and Welfare (MHLW) in Japan. LCD including 0.63% calcium was made on the basis of actual calcium consumption in Japanese. The SCD containing LCD and the newly developmental snack, was supplied as a diet including calcium content same as NCD. The newly developmental snack was composed of calcium, magnesium, CPP and black soybean. As the mainly composition, 1.3% of calcium, 0.6% of magnesium, 0.15% of CPP and 12.8% of black soybean were included in this snack. NCD was given up to six weeks after sham operation in the group 1. Other five groups were given LCD until six weeks after gonadectomy. Six weeks after surgery, group 3 and group 5 in each sex were given SCD including the newly developed bean snack.

### Histomorphometric analysis

Eighteen weeks after surgery, all the animals were sacrified under general anesthesia and the femur was fixed with 4% formaldehyde and prepared for histomorphometric analysis. Peripheral Quantitive Computed Tomography (XCT Research SA, Stratec Medizintechnik GmbH, Pforzheim, Germany) was used to quantify bone density and bone mineral content. Femur was measured at a point 1.4 mm distal area from chondrocyte growth plate. The cortical bone area was defined as over 690 mg/cm^3 ^threshold and the trabecular bone area was defined as under 395 mg/cm^3 ^threshold.

### Statistical analyses

We performed pairwise comparisons (Fisher) to examine the difference in measured values between the groups with a confidence level greater than 95%. All the data are presented as means ± standard deviations.

## Results

**Figure 2:**
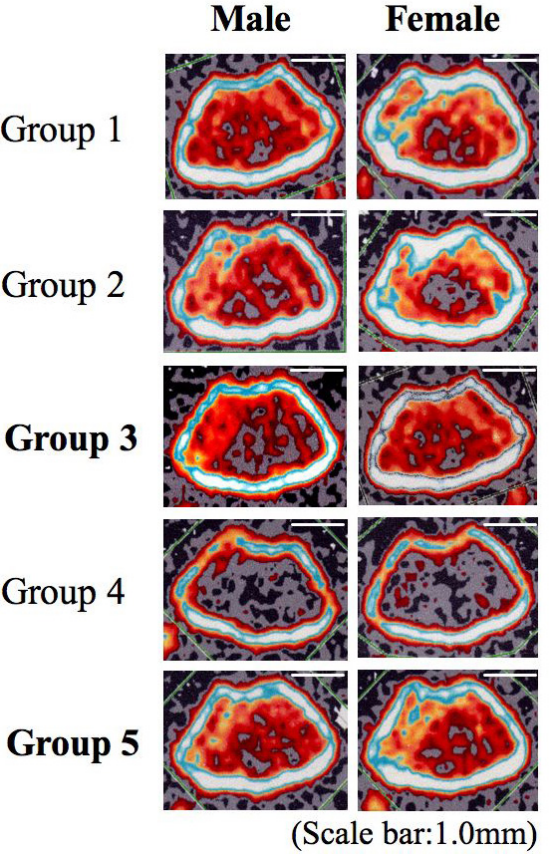
**Photographs of femur section**.

Figure 2 shows the photographs of femur section examined by pQCT at the end of experiment. Irrespective of the sex differences, the trabecular bone volume surrounded by the thick cortical bone was maintained at high level in the control groups, in sham operated mice fed NCD in particular. Meanwhile, trabecular bone volume was decreased moderately in the experimental group 2 with LCD as compared with control mice. Furthermore, the group 4, gonadectomized mice fed LCD exhibited an excessive decrease in bone volume as compared with the groups 1 and 2. On the other hand, the femur section in the group 5, osteoporosis model mice given SCD, presented a prominent recovery of trabecular bone volume.

**Figure 3:**
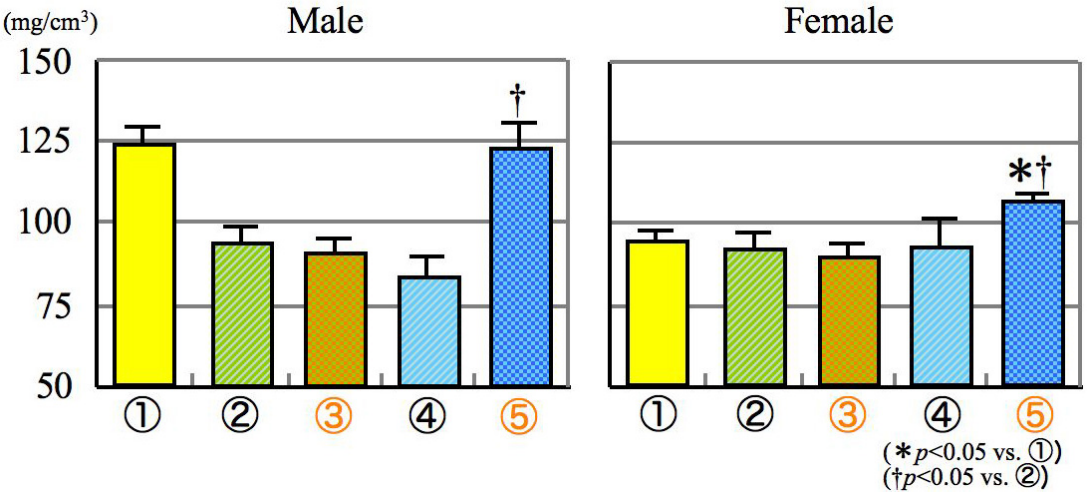
**Trabecular bone density**.

As a result of pQCT analysis, similar phenomena were observed as mentioned above for the femur structures. The trabecular bone density of gonadectomized mice fed SCD was significantly increased as compared with sham opereated mice given LCD in both genders (Figure 3). Moreover, the bone density was also significantly increased only in the female mice of group 5 as compared with sham operated mice fed NCD.

**Figure 4:**
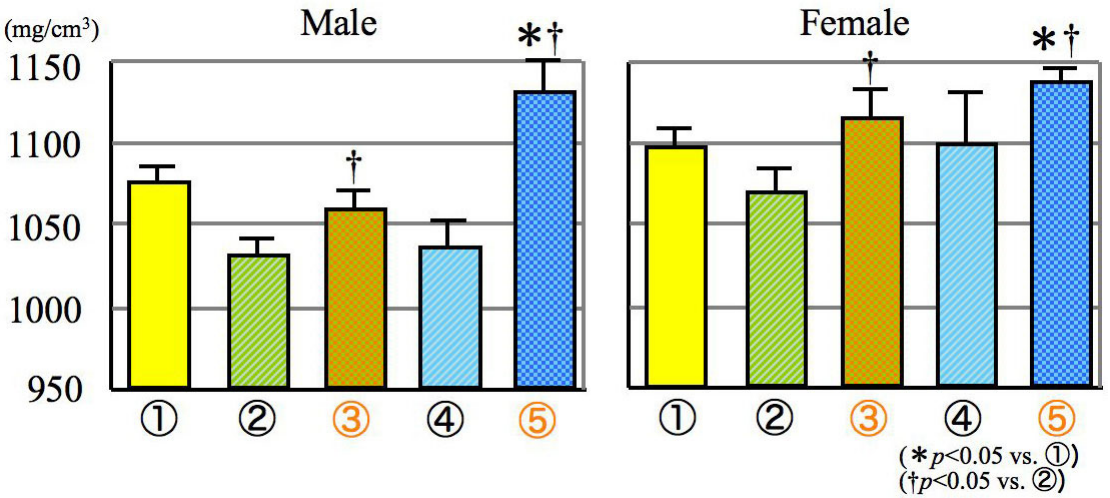
**Cortical bone density**.

For the cortical bone density, different findings from above mentioned results were obtained. Improvement effect was revealed in group 5, experimental mice given SCD and group 3, sham operarted mice fed SCD (Figure 4). These tendencies were observed in both sexes.

**Figure 5:**
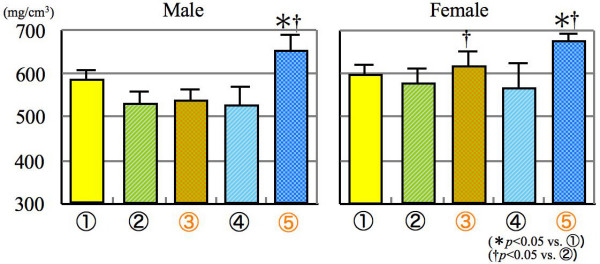
**Total bone density**.

**Figure 6:**
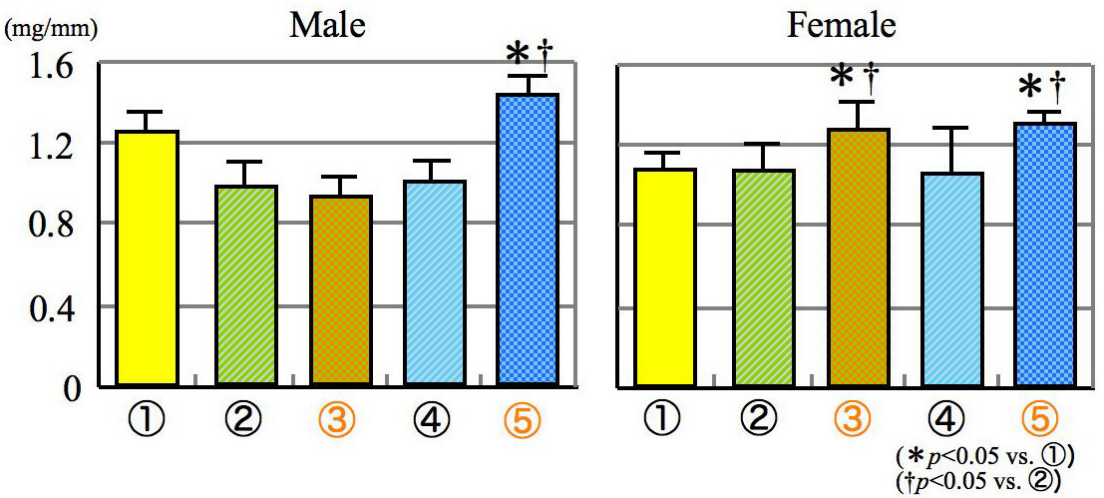
**Bone mineral content**.

Subsequently, the total bone density was apparently increased as compared with other sham operated mice. The reason may be due to SCD supplied to the gonadectomized mice, especially in the female mice (Figure 5). For bone mineral content, the groups 3 and 5 mice given SCD exhibited a significant increase as compared with the groups 1 and 2, especially in female mice. It is demonstrated from these findings that the newly development snack is very effective for the improvement of reduced bone quality or cure of osteoporosis (Figure. 6).

## Discussion

This snack we developed was composed of calcium, magnesium, CPP and black soybean. We have examined into body weight and height of all mice during experimental period. However, all mice of supplied food in these measurements were no significant difference compared with the control groups in both sexes. In addition, the amount of all nutrition including supplied food was restrained according the instructions of MHLW in Japan. Therefore, we assumed that supplied special diet food has no occurrence of side effect following snack administration.

It is well known that calcium intake decreases the risk of bone fracture[13]. It was also reported that the calcium supplementation suppress bone formation when magnesium is deficient[14]. Based on these reports, we designed to mix calcium and magnesium at an appropriate proportion of 2 to 1. These contents in this snack were compounded to fulfill ninety percentage of the daily-required nutrition defined by MHLW in Japan.

It was also reported that addition of CPP will have a beneficial effect on the absorption of calcium[15]. In Japan, CPP was accredited as a food for qualified health uses by MHLW. Accordingly, we add a certain amount of CPP to absorb mineral content easily.

The black soybean contains isoflavone which is widely accepted to have a weak estrogen activity, and to be able to bind estrogen receptor. Actually, Brandi (2003) and Miyauchi and others (1996) reported that isoflavone had either effect for the suppression of bone resorption or enhancement of bone formation, affecting directly both osteoclasts and osteoblasts [16,17]. In addition, isoflavone was revealed to affect bone metabolism similarly to the sex hormone-like effect in male[18]. Recently, it was also reported that isoflavones have revealed inhibition of bone loss in castrated male mice and growing male mice respectively [19-21]. Under such background, a new snack was developed by use of black soybean. The amount of isoflavone in this snack was determined with a special reference to the amount of safety surplus nutrition per day defined by MHLW.

Decrease in the trabecular and cortical bone volumes after gonadectomy has already been demonstrated by our previously reports [22,23]. We also reported that bone growth was significantly suppressed in the gonadectomized mice immediately after birth [24,25]. Moreover, we clarified that decrease in bone volume was occurred four weeks after gonadectomy. According to these results, we examined bone density of the femur six weeks after ovariectomy and orchiectomy.

The density of trabecular and cortical bones in the gonadectomized mice given LCD was significantly lower than in the sham operated mice given NCD and LCD, respectively. It was revealed that the deficiency of calcium intake caused decrease in bone density, under sex hormone disturbances in particular. Therefore, it is speculated that the bone density is below the optimal level in Japanese and fracture risk may become higher for aged people. These findings also support that the incidence of primary osteoporosis is higher in Japan than in American and European countries.

The bone metabolism is classified into two types, high turnover type to accelerate both bone formation and resorption and low turnover type caused by degradation of bone formation. In this study, twelve-week-old mice with sham operation fed LCD are regarded as young growing humans with low turnover type, whereas gonadectomy mice to simulate sex hormone disturbance in the experimental groups are assumed to be under the condition of postmenopausal osteoporosis as a high turnover type. Irrespective of the turnover types, however, the bone density in the group 5 given appropriate amount of calcium by supplying SCD exhibited a remarkable increase. It is thus suggested that sufficient calcium quantity through nutrition of newly developed bean snack enhanced bone formation irrespective of age.

It is reported that postmenopausal women with daily calcium intake of less than 400 mg experience significant bone loss and that calcium intake of 800 mg per day is effective for improving postmenopausal bone loss [26,27]. On the other hand, it is well known that improvement effect against bone volume loss by calcium intake is available only at the initial stage of treatment. In addition, Riggs clarified that the effect on bone loss is weaker than those reported for estrogen and bisphosphonates therapy, indicating that calcium supplements alone can't substitute treatment for osteoporosis[28]. In this study, longitudinal effect of newly developmental snack intake was not examined. However, It is hopefully anticipated that new bean snack could contributed to the enrichment of QOL as a nutrition function food, because of it was contain of several ingredients to promote assimilation efficiency of calcium.

## Conclusion

The new snack we developed included proper amount of calcium, magnesium, CPP and black soybean. When this product was given to the osteoporosis model mice, bone density of the femur was significantly increased. From these results, it is suggested that this product supplement promote bone formation irrespective of gender and age. We demonstrated that newly developmental snack supplements may be a useful preventive measure for Japanese whose bone mineral density values are less than the ideal condition.

## Competing interests

The authors declare that they have no competing interests.

## Authors' contributions

JO, RAMH, TF and KT conceived of and designed the study. JO and HS performed the statistical analyses. JO, TK, MK, MM, NT, HK, YM, HH, SA and KT interpreted the results. JO drafted the manuscript. All authors revised the manuscript for intellectual content, and read and approved the final manuscript.
